# Bone loss in patients with the ileostomy and ileal pouch for inflammatory bowel disease

**DOI:** 10.1093/gastro/got030

**Published:** 2013-11-13

**Authors:** Supriya Gupta, Bo Shen

**Affiliations:** Digestive Disease Institute, Cleveland Clinic, Cleveland, Ohio

**Keywords:** Bone loss, Crohn’s disease, inflammatory bowel disease, ulcerative colitis

## Abstract

Low bone mineral density (BMD) or low bone mass is common in patients with inflammatory bowel disease (IBD). Studies have shown that low BMD is also common in patients with ulcerative colitis (UC) even after colectomy and ileal pouch–anal anastomosis (IPAA). The reported frequency of osteopenia ranged from 26–55% and that of osteoporosis ranged from 13–32% in patients with IPAA. Increasing age, low body mass index, lack of calcium supplementation and high inflammatory activity with villous atrophy in the ileo-anal pouch are risk factors for low bone mass in pouch patients. Bone loss is also common in patients with IBD and ostomy. Current professional society guidelines do not specifically address the need for surveillance in patients with ileal pouches or ostomy. A growing body of evidence suggests that patients with ileal pouch or ostomy are at an increased risk of bone loss. Pending prospective studies, screening and surveillance using dual energy X-ray absorptiometry (DEXA) along with calcium/vitamin D supplementation may be beneficial in those patients.

## INTRODUCTION

Ulcerative colitis (UC) and Crohn’s disease (CD) are two common forms of chronic inflammatory bowel disease (IBD), in whom bone loss is common. In addition, fractures secondary to osteoporosis are associated with significant morbidities and health care costs. The current American College of Gastroenterology (ACG), American Gastroenterological Association (AGA) and European Crohn’s and Colitis Organization (ECCO) guidelines recommend screening for bone loss using dual-energy X-ray absorptiometry (DEXA) in patients with CD or UC who have a history of smoking, low body mass, family history of osteoporosis, nutritional deficiencies, hypogonadism, age >60 years, active inflammation, the current or prior use of corticosteroids for >3 months, postmenopausal state, and history of fragility fractures [1–6].

Studies on the natural history of IBD showed that 60% of patients with CD and 15%–30% of patients with UC require surgical intervention for the management of their disease [7, 8]. Restorative proctocolectomy with ileal pouch–anal anastomosis (IPAA) is the surgical treatment of choice in patients with medically refractory UC or UC with neoplasia [9]. This procedure is associated with an improvement in quality of life, due to maintenance of gastrointestinal tract continuity and reduction in dose or discontinuation of corticosteroids and other IBD-related medications. It is unclear whether restorative proctocolectomy with IPAA has a protective or detrimental effect on bone mineral density (BMD) in patients with UC. Prior studies have shown that low BMD is common in patients with UC, even after surgery [10]. Similarly, emerging data has shown a low BMD in patients with CD after surgery and ileostomy [11, 12]. Current ACG and AGA guidelines do not have specific recommendations regarding screening for bone loss in UC or CD patients after surgery. The purpose of this review is to summarize the existing literature on bone loss in IBD patients after surgical treatment and to discuss the implications for its screening and management.

## BONE LOSS IN INFLAMMATORY BOWEL DISEASE

### Incidence and prevalence

Patients with IBD have a higher risk of developing bone loss than the general population. The reported frequency of osteopenia and osteoporosis in IBD ranged from 22 to 77% and from 17 to 41%, respectively [13–15]. The risk of fracture is 40% higher in patients with IBD than that in the general population [16]. CD and UC appear to have a similar risk for fracture [2]. In a population-based study, the incidence of bone fracture was 86.2 per 10 000 patients with CD and 112.4 per 10 000 patients with UC [16]. In a separate population-based study, the incidence of bone fracture in patients with CD was 36% at the 20-year follow up and 40% in patients with UC at the 25-year follow up [17, 18] (Table 2).

### Risk factors and mechanisms for bone loss in IBD

The etiology of low bone mass in IBD patients is probably multifactorial. Reported risk factors include increasing age, long-term (>3 months) or recurrent corticosteroid use, persistently active disease, long disease duration, prior history of osteoporotic fracture, low body mass index (BMI), malnutrition, hypogonadism, lack of exercise or supplementation of calcium and vitamin D, immobilization, and smoking [2, 4, 5, 13, 19–21]. Long-term glucocorticosteroid use is a well-known cause of osteoporosis, resulting from increased osteoclastic activity. Excessive pro-inflammatory cytokines, such as tumor necrosis factor-alpha (TNF-α) and interleukin-6 (IL-6) in the setting of IBD have been shown to increase osteoclastic activity, resulting in bone resorption [22]. Therapy with anti TNF- α biologics, such as infliximab, was associated with increased bone formation without an increase in bone resorption in patients with CD, supporting the role of excessive inflammatory cytokines in bone loss [22].

### Recommendations

DEXA has been the ‘gold standard' for measuring the bone mass. This technique is widely used to characterize fracture risk in large epidemiological studies. Measurements are usually obtained at the femoral neck (hip) and lumbar spine. These measurements are reported in the form of a Z score, i.e. number of standard deviations (SD) above or below the mean for an age-matched population or T-score, which is the number of standard deviations above or below the mean for a young adult at peak bone density [1]. The World Health Organization (WHO) defines osteoporosis as T-score at the hip or spine < -2.5, osteopenia as T-score between -1 and -2.5 and normal bone density as T-score better than -1, in postmenopausal women. In contrast, the International Society for Clinical Densitometry proposed two categories: low and normal bone mass. The low bone mass was defined as a T-score of the lumber spine, total hip, or femoral neck of -2.5 or less in post-menopausal women and in men 50 years of age and older; or a Z-score of -2.0 or lower in women before menopause or men younger than 50 years [23]. The current AGA and ACG guidelines recommended screening for bone loss in IBD patients with a high risk features as mentioned above [1, 3, 4]. Additionally, blood tests, such as complete blood count, alkaline phosphatase, calcium, creatinine, testosterone (in males), 25-OH vitamin D and protein electrophoresis are recommended. The ECCO guidelines recommended screening for bone loss using DEXA in those IBD patients with persistently active disease or with repeatedly exposed to corticosteroids, or with a long disease duration [5, 6].

## BONE LOSS IN PATIENTS WITH ILEAL POUCHES

### Frequency

Studies evaluating bone loss in IBD patients after surgery are summarized in Table 1. The reported frequency of osteopenia in UC patients after IPAA ranged from 26 to 55% [24–26], and that of osteoporosis ranged from 13 to 32% [10, 25–27]. The reported incidence of fragility fractures ranged from 7 to 15% in these studies [10, 25, 27], implying that these patients are at high risk for low BMD-related complications after IPAA. In the study by Navaneethan *et al.* [27], comparing BMD in UC patients with (*n = *267) and without (*n = *119) IPAA, fragility fractures were noted more frequently in the IPAA group than in the UC group (8.1% vs 2.5%, *P = *0.038).These findings imply that proctocolectomy may not be protective from bone fracture in patients with UC.

**Table 1.** Studies evaluating bone loss in ulcerative colitis after colectomy

Study	Study design	Cases	Controls	Results
Shen B *et al.* [10]	Cross sectional, case-control	105 UC + IPAA patients with low BMD	222 UC + IPAA patients with normal BMD	32% of UC + IPAA patients had a low BMD.
				30% of patients with normal pouch or irritable pouch syndrome had a low BMD.
				Risk factors for bone loss: advanced age, low BMI, non-use of calcium supplement
Navaneethan U *et al.* [27]	Cross sectional, case-control	267 UC + IPAA patients	119 UC patients without colectomy	Low BMD more common in UC + IPAA patients than UC patients (31 vs 15%; *P = *0.001).
				Risk factors for bone loss: advanced age, low BMI and the presence of IPAA
Kuisma J *et al.* [24].	Case series	88 UC + IPAA patients and 20 UC + ileostomy patients	NA	Low BMD more common in pouch patients with sub-total or total villous atrophy than those with normal villous structure (37 vs 0%).
				The frequency of osteopenia and osteoporosis was 26.1 and 2.3%, respectively in patients with UC and IPAA.
				The frequency of osteopenia and osteoporosis was 30 and 5%, respectively in patients with UC and ileostomy.
				The lowest BMD was seen in patients with inflammation in the afferent limb
Abitbol V *et al.* [25]	Case series, longitudinal	20 UC + IPAA patients	NA	Osteopenia (55%) and vertebral crush fractures (15%) common in UC + IPAA. Spontaneous increase in BMD (7 out of 15 patients) over time (mean 28 months) was seen after pouch surgery.
McLaughlin SD *et al.* [26]	Case series	53 UC + IPAA patients	NA	The frequency of osteopenia and osteoporosis was 43 and 13%, respectively

**Table 2.** Studies evaluating bone loss in patients with ostomy

Study	Study design	Cases	Controls	Results
De Jong DJ *et al.* [11]	Case series	91 patients with CD, of which 59 patients (65%) had stoma.	NA	The frequency of osteopenia and osteoporosis was 50 and 30%, respectively.
				On multivariate analysis, BMI and history of bowel resection were found to be significant predictors for low BMD.
Robinson RJ *et al.* [12]	Cross-sectional study	117 patients with CD, of which 76 patients (65%) had stoma	NA	The frequency of osteopenia and osteoporosis was 29 and 11%, respectively.
				Patients with previous bowel resection had significantly reduced BMD, irrespective of site of surgery.
Kuisma J *et al.* [24]	Case series	20 patients with UC and ileostomy.	NA	30% of patients had osteopenia
Gupta S *et al.* [42]	Case-control study	37 IBD + Ostomy with low BMD	89 IBD + Ostomy with normal BMD	29.4% of IBD + patients ostomy had a low BMD.
				Fragility fracture was 5 times more common in patients with IBD + ostomy.

Few studies have evaluated the change in BMD over time after IPAA. In a study by Jensen *et al.* evaluating 24 patients with UC undergoing IPAA, BMD increased by a mean of 1.6% over 4–6 years after IPAA [28]. In a longitudinal study of 15 patients with IPAA, 7 (47%) were found to have an increase in BMD over a mean of 28-month follow up [25]. Similarly, in the study of 267 pouch patients by Navaneethan *et al.*, 13 patients had a longitudinal follow-up on BMD [27]. In that study, 7 out of 13 patients (54%) were found to have an improvement in BMD after a median of 46 months. These studies highlight that some patients have an improvement in BMD after proctocolectomy with IPAA, which may be related to removal of the diseased colon, discontinuation of corticosteroids and improvement in nutritional status. This may imply that proctocolectomy with IPAA may have some protective role on BMD in some (not in other) patients with IBD. The finding of severe osteopenia/osteoporosis in only 2.3% of patients after IPAA in a cohort study by Kuisma *et al.* further supports the notion [24].

Therefore, whether or not the IPAA procedure improves BMD in patients with IBD is controversial. On multivariate analysis, after adjusting for steroid use and severity and duration of disease in our previous study the presence of IPAA was significantly associated with a low BMD with odds ratio 6.02 (95% CI 2.46–14.70) [27]. Prospective studies are needed to further elucidate the association between bone mass and pouch surgery.

### Vitamin D and calcium absorption in the gut

The two main forms of vitamin D are vitamin D2 (ergocalciferol) and vitamin D3 (cholecalciferol). Vitamin D from plant sources is in the form of vitamin D2 and that from animal sources is vitamin D3. Vitamin D3 is also produced in the human skin by 7-dehydroxycholesterol after absorption of ultraviolet B light from the sun. Vitamin D from dietary sources is incorporated into chylomicrons and absorbed, mostly from the proximal small bowel, and transported via the gut lymphatics into the venous circulation after being bound to vitamin D binding protein, an alpha-globulin produced in the liver. Vitamin D is then hydroxylated in the liver by cytochrome P450-like enzymes to form 25-hydroxyvitamin D [25(OH)D], which is the major circulating and storage form of vitamin D. Further hydroxylation of vitamin D to form 1,25-dihydroxyvitamin D [1,25(OH)2D] occurs in the kidney. This last step in the kidney is stimulated by parathyroid hormone (PTH), whereas calcium and 1,25(OH)2D itself inhibits it.

Calcium absorption via the intestine occurs via active (transcellular) and passive (paracellular) processes. Active absorption, which is the main mechanism, is controlled by 1,25 (OH)2D. Active calcium absorption also occurs mainly in the proximal small bowel; however some calcium absorption occurs in other segments of the small bowel. Optimal calcium absorption requires the presence of gastric acid.

### Risk factors and possible mechanisms of bone loss

#### Vitamin D malabsorption

Formation of IPAA after proctocolectomy alters the normal anatomy and physiology of the small intestine. Vitamin D is a fat-soluble vitamin, while bile salt metabolism is important for lipid absorption. Bacterial overgrowth leads to deconjugation of bile salts, leading to formation of free bile acids. This impairs the formation of bile-salt-lipid micelle complexes, which leads to dietary fat malabsorption, which in turn can lead to vitamin D deficiency. It has been shown that stasis of stool in the ileum in patients with UC and IPAA causes bacterial overgrowth that causes deconjugation of bile salts leading to malabsorption of vitamin D [29–31]. Our previous study showed that the lack of calcium supplementation was found to be a predictor of low BMD in patients with UC and IPAA [10]. In another study, low vitamin D levels were more prevalent in patients with UC and IPAA than in UC without IPAA (69% vs 42%) [32]. Vitamin D receptor (VDR) may play an important role in maintaining gastrointestinal mucosal integrity, as VDR-knockout mice have been shown to develop severe colitis [33]. However, there appears to be no association between a low vitamin D level and pouch inflammation [32]. Other hypotheses include involvement of the proximal small intestine in patients with UC. One study has shown the presence of diffuse duodenitis in some patients with UC [34]. Patients with UC and IPAA have been shown to have evidence of inflammation in the duodenum on endoscopy, as well as histologically [35, 36]. These changes in the proximal small intestine—the site of vitamin D and calcium absorption—can lead to malabsorption and hence vitamin D deficiency. Prospective controlled studies are needed to evaluate vitamin D levels in patients before and after IPAA, to understand the mechanism of ongoing bone loss in these patients.

#### Pouch inflammation

It has been shown that with the formation of IPAA, villous atrophy occurs due to fecal stasis, bacterial overload, inflammation, which may also contribute to osteopenia and osteoporosis. The study by Kuisma *et al.*, consisting of 88 patients with IPAA, found that 37% of patients with sub-total or total villous atrophy had osteopenia, as compared to 0% of patients with IPAA and normal villous structure [24]. In addition, these patients with sub-total or total villous atrophy had a threefold higher lifetime corticosteroid dose than those with normal villous structure, indicating greater inflammatory activity at baseline in those patients. Presence of inflammation is associated with circulating cytokines, such as interleukins (IL-1, IL-6) and TNF-α, which stimulate osteoclast activity and lead to bone resorption and bone loss [37]. In the study by Kuisma *et al.*, patients with osteopenia suffered more exacerbations of pouchitis, highlighting the role of inflammation in bone loss after IPAA [24]. In contrast, the study from our institution, evaluating 327 patients with UC and IPAA from subspecialty Pouchitis Clinic who underwent DEXA, found that the presence of inflammatory pouch conditions (such as chronic pouchitis, CD of the pouch, or cuffitis) were not predictive of low BMD in patients with IPAA in multivariable analysis [10]. However, in that study, chronic inflammation of the pouch was significantly more prevalent in patients with low BMD than in those with normal BMD in univariate analysis (66% vs 44%; *P < *0.001). These studies highlight the potential role of ongoing inflammation in bone loss in patients with IBD and IPAA.

The exact mechanisms of bone loss in IPAA patients are not clear. We found that IPAA procedure was associated with various adverse metabolic consequences, such as chronic anemia [38], vitamin D deficiency [32], and renal stones [39], along with bone loss. Surprisingly, those adverse metabolic consequences were not shown to be related to chronic inflammation of the pouch and they can also occur in patients with normal pouches or irritable pouch syndrome. This phenomenon suggests that the bowel anatomy-altering pouch procedure may disrupt the luminal ecosystem, leading to various metabolic abnormalities. Similar phenomena have been observed in other bowel-altering surgical procedures, such as gastric bypass surgery [40, 41].

### Recommendations

UC patients with IPAA have not been recognized as at high risk for bone loss in the current guidelines. Consequently there are no guideline recommendations for screening for BMD in patients with IPAA [3]. In the light of the accumulating evidence discussed above, we recommend that these patients with IPAA should be considered as a high-risk group for bone loss. Baseline DEXA prior to surgery and close monitoring of BMD after IPAA should be considered in addition to calcium and vitamin D supplementation in these patients.

## BONE LOSS IN PATIENTS WITH STOMA

### Frequency

Bone loss in IBD patients with bowel resection has been evaluated. In a study of 117 CD patients, of whom 65% had history of prior bowel resection, the frequency of osteopenia and osteoporosis was 29% and 11%, respectively [12]. Similarly, in a separate study of 91 CD patients, of whom 65% had prior history of bowel resection, the frequency of osteopenia and osteoporosis was 50% and 30%, respectively [11]. In patients with UC and ileostomy, Kuisma *et al.* found that 30% of patients had osteopenia [24]. In 126 patients with IBD and ostomy, our group reported that 29.4% of patients had a low BMD [42].

### Risk factors and possible mechanisms

Possible risk factors for bone loss in patients with IBD and ostomy include malabsorption secondary to bowel resection, malnutrition and more aggressive disease and inflammation which led to bowel resection in the first place. In a study evaluating 126 patients with IBD and ostomy, we found that low BMI and history of fragility fracture were predictors of low BMD in these patients [42]. Further studies are needed to evaluate risk factors for bone loss in patients with IBD and ostomy. The current guidelines do not characterize patients with IBD—and ostomy in particular—as at high risk for bone loss.

### Recommendations

The current guidelines do not recognize patients with IBD and ostomy as a high risk group for low BMD. The European Society for Parenteral and Enteral (ESPEN) guidelines recommend screening for BMD using DEXA on a yearly basis in patients on total parenteral nutrition (TPN) [43]. The ACG, AGA, ECCO and American Society for Parenteral and Enteral (ESPEN) guidelines do not specifically recommend DEXA in patients with IBD and ostomy. Further studies evaluating bone loss in patients with IBD and ostomy are needed.

## CONCLUSIONS

Low BMD is common in patients with IBD with IPAA and ileostomy. Increasing age, low BMI, lack of calcium supplementation and high inflammatory activity with villous atrophy in the ileo-anal pouch are risk factors for low BMD in these patients with IPAA. Patients with IPAA should be considered at high risk for bone loss and fractures. Pending prospective studies, baseline and follow up DEXA screening along with calcium/vitamin D supplementation may be beneficial in these patients.

## FUNDING

This project was partially supported by the Ed and Joey Story Endowed Chair (to B.S.).

**Conflict of interest:** none declared.
